# Statement of Retraction: Long non-coding RNA DCST1-AS1/hsa-miR-582-5p/HMGB1 axis regulates colorectal cancer progression

**DOI:** 10.1080/21655979.2023.2299524

**Published:** 2024-02-20

We, the Editors and Publisher of the journal Bioengineered, have retracted the following article:

Long huang & Gang Dai (2022) Long non-coding RNA DCST1-AS1/hsa-miR-582-5p/HMGB1 axis regulates colorectal cancer progression, Bioengineered, 13:1, 12-26, DOI: 10.1080/21655979.2021.1976894

Since publication, significant concerns have been raised about the integrity of the data and reported results in the article. When approached for an explanation, the authors did not respond, nor did they provide their original data or any necessary supporting information.

As verifying the validity of published work is core to the integrity of the scholarly record, we are therefore retracting the article. All authors listed in this publication have been informed.

We have been informed in our decision-making by our editorial policies and the COPE guidelines.

The retracted article will remain online to maintain the scholarly record, but it will be digitally watermarked on each page as ‘Retracted’.

